# Toxicology and Drug Delivery by Cucurbit[n]uril Type Molecular Containers

**DOI:** 10.1371/journal.pone.0010514

**Published:** 2010-05-06

**Authors:** Gaya Hettiarachchi, Duc Nguyen, Jing Wu, Derick Lucas, Da Ma, Lyle Isaacs, Volker Briken

**Affiliations:** 1 Department of Cell Biology and Molecular Genetics, University of Maryland, College Park, Maryland, United States of America; 2 Department of Chemistry and Biochemistry, University of Maryland, College Park, Maryland, United States of America; Universität Heidelberg, Germany

## Abstract

**Background:**

Many drug delivery systems are based on the ability of certain macrocyclic compounds – such as cyclodextrins (CDs) – to act as molecular containers for pharmaceutical agents in water. Indeed β-CD and its derivatives have been widely used in the formulation of hydrophobic pharmaceuticals despite their poor abilities to act as a molecular container (e.g., weak binding (K_a_<10^4^ M^−1^) and their challenges toward chemical functionalization. Cucurbit[n]urils (CB[n]) are a class of molecular containers that bind to a variety of cationic and neutral species with high affinity (K_a_>10^4^ M^−1^) and therefore show great promise as a drug delivery system.

**Methodology:**

In this study we investigated the toxicology, uptake, and bioactivity of two cucurbit[n]urils (CB[5] and CB[7]) and three CB[n]-type containers (Pentamer **1**, methyl hexamer **2**, and phenyl hexamer **3**). All five containers demonstrated high cell tolerance at concentrations of up to 1 mM in cell lines originating from kidney, liver or blood tissue using assays for metabolic activity and cytotoxicity. Furthermore, the CB[7] molecular container was efficiently internalized by macrophages indicating their potential for the intracellular delivery of drugs. Bioactivity assays showed that the first-line tuberculosis drug, ethambutol, was as efficient in treating mycobacteria infected macrophages when loaded into CB[7] as when given in the unbound form. This result suggests that CB[7]-bound drug molecules can be released from the container to find their intracellular target.

**Conclusion:**

Our study reveals very low toxicity of five members of the cucurbit[n]uril family of nanocontainers. It demonstrates the uptake of containers by cells and intracellular release of container-loaded drugs. These results provide initial proof-of-concept towards the use of CB[n] molecular containers as an advanced drug delivery system.

## Introduction

The improvement of public health relies in large part upon the discovery and approval of new drugs. Unfortunately, in recent years only about 8% of compounds submitted for clinical development are approved compared to nearly 14% ten years ago [1]. Studies have shown that one major reason for this decreased success rate is poor drug bioavailability [2]. Bioavailability is defined as the rate and extent to which the active ingredient in a drug formulation becomes available at the site of necessary action [3]. Factors that influence drug bioavailability are solubility, *in vivo/in vitro* stability, ability to cross internal membranes, toxicity, distribution and/or metabolism among other factors. Each aspect of drug bioavailability is key during the drug discovery process [3], therefore if adequate solutions to low bioavailability are not devised, further development of a drug candidate is unlikely. Because more and more drug candidates are failing to meet acceptable standards of bioavailability the number of novel, commercially available drugs is decreasing, while the funds invested in the drug discovery process are increasing [2]. For this reason, extensive interest has turned towards the approach of improving the bioavailability of drug candidates via the use of drug delivery vehicles [2].

One approach to improve the bioavailability of drug candidates is to non-covalently encapsulate them within molecular containers. To date a number of classes of molecular containers (e.g. dendrimers, cyclodextrins (CDs), and nanoparticles) have shown promise in improving drug bioavailability. For example, dendrimers are globular structures that are composed of repeated branches forming a hollow interior which allows for the encapsulation of guest molecules. These globular complexes have been utilized in cancer treatment, wound healing, and in the prevention of HIV transmission [4], [5], [6]. Likewise, CDs, are a class of macrocyclic molecular containers that have been extensively studied for their use in drug delivery [7]. These compounds have also been demonstrated to increase drug solubility in water and enhance the absorption of anticancer drugs [8]. In summary, drug delivery systems may improve drug bioavailability by altering the solubility of a drug in water, stability during storage or *in vivo*, toxicity, side effects, drug resistance, absorption and also by providing specific targeting options to specify distribution and thus improve drug efficiency [2], [9], [10].

Over the past decade a new class of molecular containers known as cucurbit[n]urils (CB[n]) have been studied intensively because their intriguing recognition properties render them prime components for academic applications including the development of chemical sensors and molecular machines and also due to their potential to supplant the cyclodextrins as an effective drug delivery system. For example, CB[n] compounds are available in a variety of sizes (n = 5, 6, 7, 8, 10) [11] that span and exceed those available in the cyclodextrin series (α-, β-, and γ–CD; n = 5, 6, 7). These CB[n] are readily synthesized on a kilogram scale by the condensation reaction of glycoluril with formaldehyde under acidic conditions [12]. CB[5], CB[6], CB[7], and CB[8] are even commercially available. CB[n] compounds are expected to be particularly advantageous in drug delivery studies because they bind their targets strongly (generally K_a_>10^4^ M^−1^) by a combination of the hydrophobic effect and ion-dipole interactions with the ureidyl C = O groups of the CB[n] portals [13], [14], [15]. Even more importantly, the release of the guest may be triggered by a variety of stimuli (e.g. pH, photochemistry, electrochemistry) [16].

The preclinical stage of drug discovery is an integral part of the drug discovery process and is a necessary step in the development of CB[n] type molecular containers as a novel drug delivery system. This stage of the drug discovery process involves the understanding of the candidate's specific features such as the ability to cross crucial membranes, metabolic stability and their ability to pass a series of *in vitro* and *in vivo* toxicity screens to assess safety in the human system [17], [18]. Drug toxicology is a crucial aspect of drug discovery because only about 1 out of 5,000 screened drugs are approved for medicinal use due to the fact that most drugs fail toxicology assays conducted on animals [17]. Although the analysis of the chemical and biological significance of container-drug complexes of CB[n]s with albendazole [19], platinum-based anticancer drugs [20], Vitamin B(12) [21] and antibiotics such as proflavine [22] have been reported, there is very little information reported about the toxicology of the empty CB[n] containers.

This paper focuses on providing a proof-of-principle for the use of CB[n] and CB[n]-type molecular containers in drug delivery applications. In particular, we performed a systematic investigation of the cytotoxicity of the CB[n]s. Thus, we demonstrated that CB[5], CB[7], and **1–3** are well tolerated up to doses of 1 mM by human kidney (HEK293), human hepatocyte (HepG2), and murine macrophage (RAW264.7) cell lines as monitored via cell cytotoxicity and metabolic activity assays. Furthermore, we show that CB[7] complexes are well internalized by RAW264.7 and transported to lysosomes. Finally, CB[7] can be efficiently loaded with the anti-tuberculosis drug ethambutol (EMB) and be used for treatment of macrophages infected with mycobacteria. Thus, here we provide additional evidence that several members of the CB[n] family are promising tools for drug delivery.

## Results and Discussion

### Structure and synthesis of CB[n] and CB[n]-type compounds

**Figure 1:**
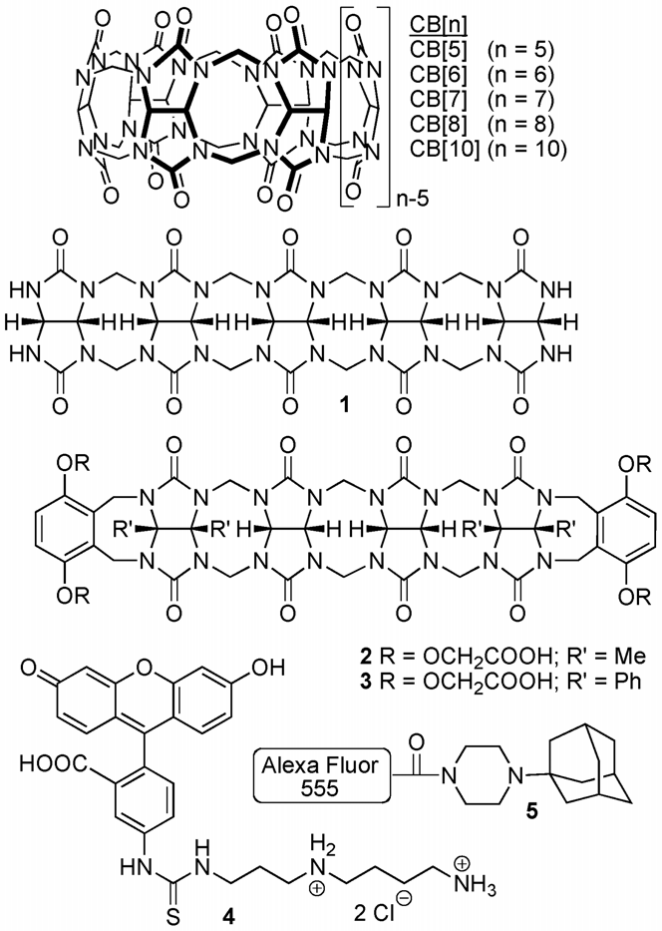
Structures of the five containers. CB7, CB5, Pentamer (**1**), Me-Hexamer (**2**), and Ph- Hexamer (**3**) and FITC (**4**) and Alexa555 (**5**) conjugates.

We selected two CB[n] compounds, CB[5] and CB[7] (Fig. 1), for this initial study because of their excellent solubility in water. From the CB[n]-type compounds available in the Isaacs lab we also selected acyclic glycoluril pentamer (**1**) and two hexamers (**2** and **3**). Compounds **1**–**3** retained the ability to bind strongly to their targets but did so with faster kinetics due to their acyclic structures [23], [24]. In order to allow for the tracking of these CB[n] molecular containers inside cells we synthesized compounds **4** [25] and **5**. Compounds **4** and **5** contain fluorescein or Alexa Fluor 555 dyes covalently attached to spermidine and adamantaneamine subunits that resulted in tight non-covalent binding to CB[n]-type compounds.

### Toxicology assays of containers using HEK 293 and RAW 264.7 cell lines

One of the major problems faced during the drug discovery process is toxicity. Therefore, there is a significant emphasis on preclinical toxicity screening predominantly through cytotoxicity assays [26], [27]. As a first step toward establishing the potential of CB[n] in drug delivery studies we decided to quantify the toxicity of CB[n] and CB[n] type containers in cellular assays. Here we use two complementary assays to analyze cytotoxicity: an MTS (CellTiter 96 AQueous Kit®) assay that measures cellular metabolism, and the AK (Toxilight®BioAssay Kit) assay that measures cell death via the release of the cytosolic enzyme adenylate kinase into the supernatant. Both assays were used with three different cell lines. Two of these cell lines, the HEK293 and HepG2 cells, are commonly used in drug toxicity studies. HEK293, a human kidney cell line, is used to assess the effect of the drug candidate on the renal system and HepG2, a human hepatocyte cell line, is used to assess the response of liver cells where drugs are metabolized. Camptothecin, erythromycin, erythromycin estolate and all five containers were used in both MTS and AK assays conducted on the HEK293, HepG2, and RAW264.7 cell lines. Erythromycin is a commercially available drug widely used to treat bacterial infections. Erythromycin estolate, however, is a derivate with high toxicity [28]. Erythromycin, with an EC_50_ value of 594 (±194) µM is significantly less toxic compared to erythromycin estolate, which has an EC_50_ of 109 (±7) µM [28]. These two drugs were chosen specifically to serve as a point of comparison for the levels of cytotoxicity resulting from the containers. Camptothecin served as a positive control since it is an anticancer drug that targets and inhibits topoisomerase I during cell division and thus induces cell death [29], [30], [31]. Hence, the use of camptothecin resulted in greater cell death is seen in RAW264.7 cells which double every 11 h [32] and less cell death is observed in HEK293 cells which replicate approximately every 24–36 h [33]. Camptothecin was not used for HepG2 cells because these cells replicate only approximately every 48 h [34], thus, making camptothecin an ineffective death inducer. Distilled water was used for HepG2 cell lysis instead.

**Figure 2:**
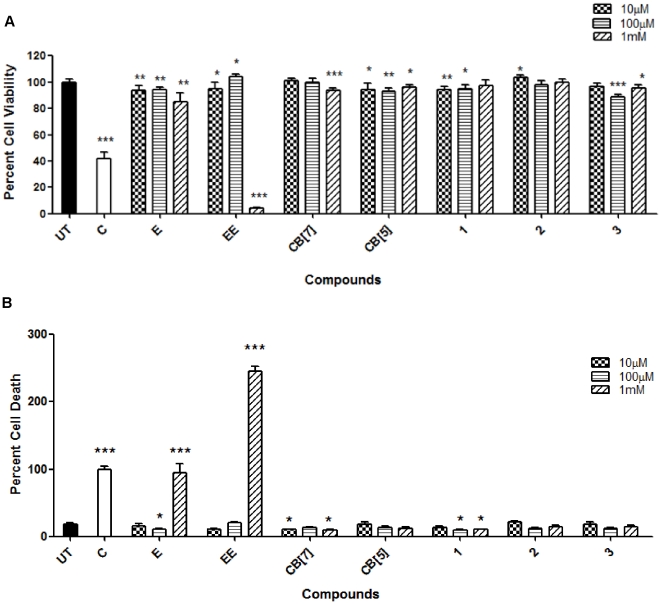
HEK293 toxicology assays. The assay was performed using CB[7], CB[5], **1**, **2**, and **3** which indicated high cell tolerance of all containers up to a concentration of 1 mM. MTS (A) and AK assays (B) performed after the cells had been incubated with indicated containers and drugs for two days (UT  =  Untreated, C =  Camptothecin, E =  Erythromycin, EE =  Erythromycin Estolate). AK assay was conducted using supernatant from cells seeded for the MTS assay. This and all other figures are representative of three replicate experiments. Statistical analysis for all figures used unpaired t-test analysis with *P = 0.01–0.05; **P = 0.001–0.01; ***P<0.001.

The MTS and AK assays for all three cell lines were conducted after two days of incubation with the containers at concentrations of 10 µM, 100 µM, and 1 mM. Relative absorbance and luminescence data was normalized to percent cell viability (MTS) and death (AK). MTS assay for HEK293 cells indicated that camptothecin treatment resulted in approximately 59% decrease in cell viability. Erythromycin, at the highest concentration of 1 mM, produced only a slight reduction in metabolic activity (84% viability) while erythromycin estolate induced an ≈25 fold decrease (4% viability). In sharp contrast containers CB[7], CB[5], and **1**–**3** at 1 mM dose resulted in 94, 96, 98, 100, and 95% cell viabilities, respectively. Consequently, the cell viability in the presence of the containers was comparable to that of the untreated cell population and was significantly higher than values for camptothecin, erythromycin and erythromycin estolate (Figure 2A). These results suggest that all CB[n]-type containers have good biocompatibility. As a complementary method to assess biocompatibility we used the AK assay that measures cell death through the release of adenylate kinase via a luminescence read-out. The relative luminescence units (RLU) values were then converted to relative cell death scale with camptothecin treatment set at an arbitrary value of 100. Thus, the untreated cell population indicated only 18% cell death and CB[7], CB[5], **1, 2,** and **3** at a concentration of 1 mM resulted 9, 11, 10, 14 and 14% cell death. In contrast, at a concentration of 1 mM erythromycin (82%) and erythromycin estolate (246%) presented higher values of cell death comparable to the camptothecin treated population (Figure 2B). Erythromycin estolate showed higher cell death than even the camptothecin control.

**Figure 3:**
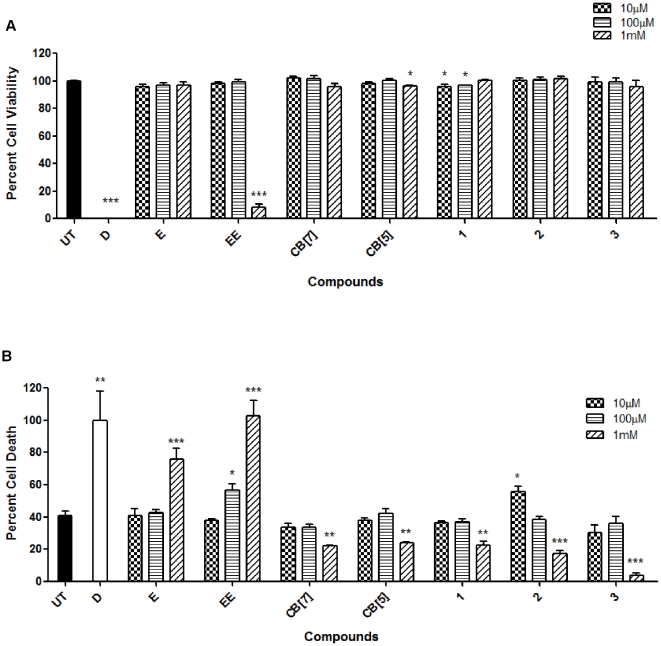
HepG2 toxicology assays using various containers. This assay showed high cell viability and low toxicity. HepG2 MTS (A) and AK (B) assays were conducted using the procedure described in Fig 2.

Toxicity studies using the MTS and AK assays for the liver cell line, HepG2, provided similar results to the HEK293 cells (Figure 3A). The MTS assay for distilled water treated HepG2 population indicated a percent cell viability of 0.28% which was comparable to that of erythromycin estolate at 1 mM (9% cell viability). However, interestingly, the cell viability for erythromycin at 1 mM indicated high survival at approximately 97%. HepG2 treatment with 1 mM of CB[7], CB[5], **1**, **2** and **3** resulted in 96, 97, 101, 102% and 96% cell viability respectively compared to 100% viability in the untreated cell population (Figure 3A). These results were also reflected in the AK assay. Untreated cells indicated only 40% cell death while erythromycin and erythromycin estolate treatment resulted in 76 and 103% relative cell death. The containers CB[7], CB[5], **1**, **2**, and **3** provided values of 22, 24, 23, 17, and 4% death respectively in the HepG2 cell line, in turn supporting the high survival values of the MTS assay and untreated cell population (Figure 3B).

**Figure 4:**
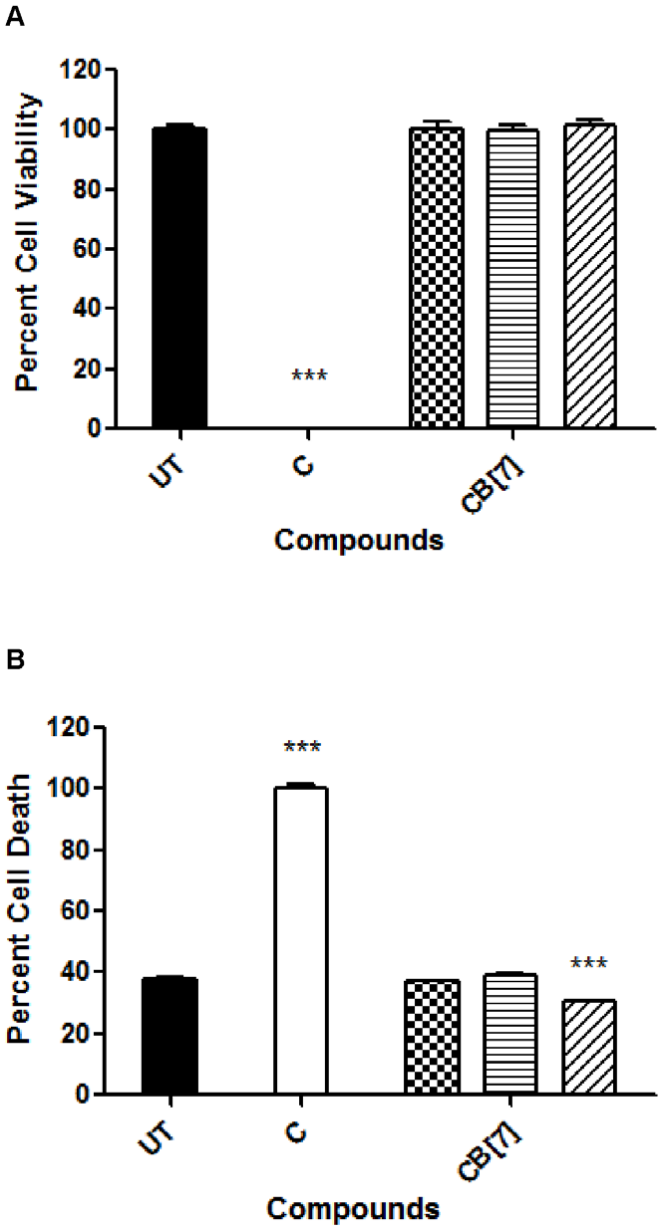
RAW264.7 toxicology assays using CB[7]. These results signified high cell tolerance at concentrations of up to 1 mM of CB[7]. RAW264.7cells were incubated with the CB[7] and camptothecin for two days before conducting both MTS (A) and AK (B) assays. Both assays were conducted following the procedure indicated in Fig 2.

*Mycobacterium tuberculosis*, the causative agent of human tuberculosis (TB), results in 200–300 million cases annually leading to 2–3 million deaths. Despite the creation of a TB vaccine and antibiotics, tuberculosis it is still an eminent problem across the globe, primarily due to emerging drug resistance and the inability of the vaccine to protect adults efficiently [35], [36], [37], [38]. A novel drug discovery system like the CB[n]-type molecular containers could prove to aid in the improvement of TB treatments [39], [40], [41]. Therefore, we started by assessing the toxicity of CB[7], the most soluble of the containers in our study, using the murine macrophage cell line, RAW264.7, via MTS and AK assays. The MTS assay for the camptothecin treated RAW264.7 cell population resulted in a decrease in cell viability by approximately 99%. At a 1 mM concentration, CB[7] was very well tolerated in the cell line producing a 101% cell survival (Figure 4A). The AK assay provided a complementary picture of the biocompatibility of CB[7] with the untreated population cell death equal to 38% whereas CB[7], at the highest dose of 1 mM, resulted in an even lower 30% cell death (Figure 4B).

In conclusion, all three cell lines supported high doses of up to 1 mM of each container, while erythromycin and more so erythromycin estolate resulted in high cell toxicity at this concentration. These results demonstrate that at least in these *in vitro* tests the CB[n] compounds are as well tolerated by eukaryotic cells as the widely prescribed antibiotic erythromycin. The low toxicity of the CB[n] compares also favorably to other nanocontainers such as liposomes, dendrimers and even cyclodextrins For example, after only one day of treatment with 0.9 mM catanionic liposomes approximately 80% cell death was observed in the macrophage cell line RAW264.7 [42]. Similarly, cationic dendrimers induced a dose-dependent cytotoxicity on RAW264.7 cells [43]. In addition, some cationic polystyrene nanoparticles induced reactive oxygen dependent cell death in RAW264.7 cells after only 16 h of incubation [44]. Cyclodextrins have been studied since the 1970s and they have shown little toxicity *in vivo*. Nevertheless, they have the capacity to extract cholesterol from eukaryotic membranes and create holes, leading to direct cytotoxicity *in vitro* in studies using erythrocytes [45]. Thus our results indicate that all of the analyzed CB[n] containers are promising drug-delivery vehicles at least in regard to their cytotoxicity.

### Uptake and localization of CB[7] using RAW 264.7 cells

Next the uptake and trafficking of fluorescently tagged CB[7] by RAW264.7 cells was analyzed. Flow cytometry was used to quantify the cellular uptake of CB[7]•**4** using a dose titration assay and a time course assay. Visual confirmation of uptake and analysis of intracellular localization was conducted using CB[7]•**5** through fluorescence microscopy.

The fluorescent CB[7]•**4** and CB[7]•**5** complexes used in these experiments are held together by non-covalent interaction. Accordingly, there is the possibility of an equilibrium between the free and bound dye forms. It is known, however, that CB[7] binds with high affinity to the adamantaneammonium ion subunit of **5** (K_a_ = 4.2×10^12^ M^−1^) and the butanediammonium ion subunit of **4** (K_a_ = 1.5×10^5^ M^−1^) [14], [46]. At the millimolar concentrations of CB[7]•**4** and CB[7]•**5** used in our experiments we calculate that at least 90% of **4** and 99% of **5** are present as their CB[7] complexes.

**Figure 5:**
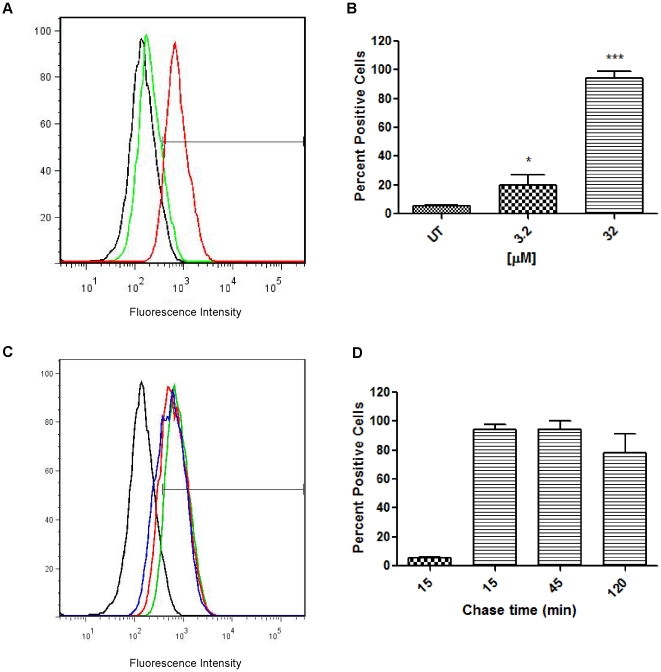
CB[7]•4 uptake and intracellular stability of CB[7] in RAW264.7 cells. Both the dose titration and time course assays used RAW264.7cells incubated with CB[7]•**4** for 20 mins prior to analysis. (A) Dose titration assay used CB[7]•**4** concentrations of 3.2 (green) and 32 µM (red). (B) Statistical analysis of the percentage of cells positive for fluorescence. (C) Timecourse assay was conducted using 32 µM of CB[7]•**4**. After incubation with the fluorescent container, cells were chased for 15 (green), 45 (red) and 120 min (blue) (D) Statistical analysis of the percentage of cells positive for fluorescence.

The dose-dependent uptake of the fluorescent CB[7]•**4** was characterized via flow cytometry using 3.2 and a 32 µM of CB[7]•**4**. CB[7]•**4** was incubated with RAW264.7 cells for 20 min before analysis. A dose of 3.2 and 32 µM resulted in median fluorescence intensity (MFI) of 197 and 703 compared to that of the untreated sample which was at a value of 131 (Figure 5A). Statistical analysis of the histograms showed the percentage of cells positive for CB[7]•**4** staining as an average of 5% for untreated cells, 24% for a concentration of 3.2 µM, and 86% for 32 µM (Figure 5B).

To determine the intracellular stability of the CB[7]•**4** complex we conducted a time course assay. CB[7]•**4** was incubated with the cells for 20 min and then chased for 15, 45, and 120 min. This resulted in high MFIs of 743 after 15 min chase, 612 after 45 min, and 544 after 120 min chase time in one representative experiment (Figure 5C). Further analysis determined that the percentage of cells positive for staining was 6% for untreated cells, 92% after 15 min chase, 91% after 45 min and finally 85% after a chase time of 120 min. This indicated that the CB[7]•**4** complex has significant intracellular stability for at least 120 min.

**Figure 6:**
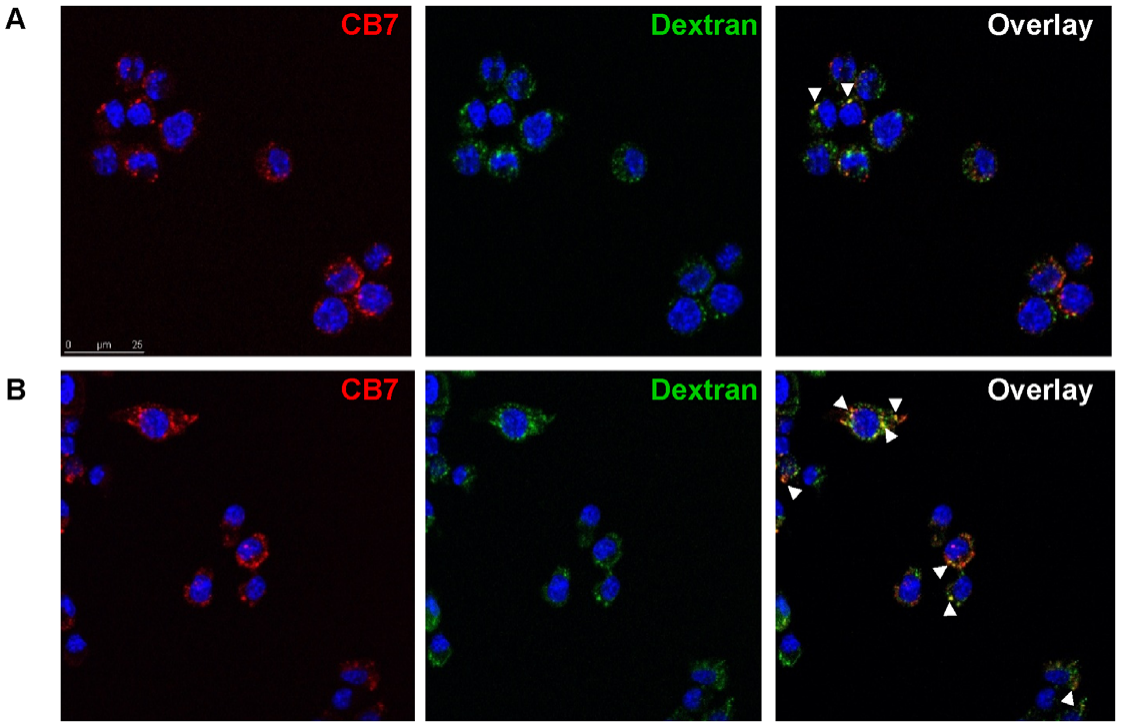
Intracellular localization of CB[7] in RAW264.7 cell. Cell incubated with Dextran-647 and CB[7]•**5** showed intracellular localization of CB[7] through the endosomal pathway. RAW264.7cells were incubated with Dextran-647 (green) overnight and CB[7]•**5** (red) for 20 min the following day. Cells were chased for 15 (A), 45 (B) and 120 min (not shown) after incubation with CB[7]•**5**. Arrows indicate co-localization.

Co-localization assays using fluorescence microscopy was conducted using Dextran-647, and CB[7]•**5** in order to analyze the intracellular localization of the container. Dextran-647 was incubated with cells at a concentration of 125 µg/mL overnight to stain cell lysosomes. Cells were then pulsed with CB[7]•**5** for 20 min and chased for 15, 45, 120 min. This analysis showed an initial uptake of CB[7]•**5** and Dextran-647 at 15 min with little co-localization (Figure 6A) however at 45 min, an increase in CB[7] co-localization with Dextran-647 was observed (Figure 6B).

CB[7] binds tightly to acridine orange and this complex was previously used to demonstrate the uptake of CB[7] by mouse muscle embryo cells (NIH/3T3) via immunofluorescence microscopy[47]. In addition, a CB[6] loaded with a FITC-spermine conjugate was created and shown to be internalized by HepG2 cells[48]. Both of these studies did not quantify the uptake of the container nor did they investigate its intracellular localization but nevertheless they demonstrated that other cell types besides the macrophages used in our study are able to take up CB[n] containers. As a whole the results of all of these studies strongly suggests that many complexes of CB[n]-type containers are able to cross the cell membrane. This ability for CB[7] complexes to cross cell membranes is of course critical if CB[n]-type molecular containers are to be used for drug delivery.

### *M. smegmatis* treatment with EMB and the CB[7]•EMB Complex

**Figure 7:**
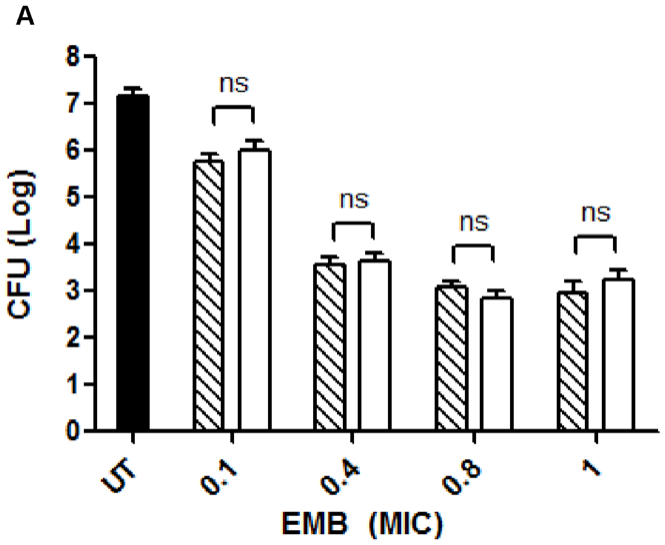
*M. smegmatis* treatment using the TB drug, EMB, and CB[7]. EMB loaded CB[7] (CB[7]•EMB, white bars) was equally effective in treating *M. smegmatis* infected RAW264.7 cells as free EMB (patterned bars). RAW264.7cells were incubated with *M. smegmatis* for two hours and then chased for three days with EMB and CB[7]•EMB. Varying MIC values for EMB and CB[7]•EMB were used: 0.1, 0.4, 0.8, and 1 units. Viable bacteria were quantified using CFU/ml. This figure is representative of two replicate experiments.

Finally, we wanted to assess what effect the loading of a drug into CB[7] would have on the bioactivity of the drug. We used *M. smegmatis*, a non-virulent mycobacterium, as a model for *in vitro* infections of macrophages. Ethambutol (EMB) is a widely used antituberculosis drug that has been associated with drug resistance in *M. tuberculosis* [35], [37], [49]. RAW264.7 cells were infected with *M. smegmatis* and then treated with EMB and CB[7]•EMB for 3 days and the amount of viable bacteria was determined (Figure 7). At day 3, untreated cells provided high bacterial survival with a CFU of 1.24×10^7^ (±5.4×10^6^) CFU/mL. At a MIC of 0.1 units, cells treated with EMB resulted in 4×10^5^ (±2.3×10^5^) CFU/mL and cells treated with CB[7]•EMB resulted in 8.6×10^5^ (±6×10^5^) CFU/mL. CFU values between EMB and CB[7]•EMB at a MIC value of 0.4 units were found to be 3.6×10^4^ (±1.7×10^3^) CFU/mL and 4.3×10^4^ (±1.7×10^3^) CFU/mL respectively. At a MIC of 0.8 units, EMB was 1×10^3^ (±4×10^2^) CFU/mL and CB[7]•EMB was 6×10^2^ (±3.3×10^2^) CFU/mL. Finally at a MIC of 1 units, CFU values for EMB and CB[7]•EMB were 8×10^2^ (±6.6×10^2^) CFU/mL and 1.4×10^3^ (±1.1×10^3^) CFU/mL respectively. The values of CFUs within each MIC dose for free EMB or CB[7]•EMB were not significantly different from each other (unpaired t-test) indicating no inhibition of the container on the ability of the drug to kill the bacteria (Figure 7).

Previously, the Kim group loaded the anticancer drug, oxaliplatin into CB[7] and showed that the cytotoxic activity of the drug was reduced by 5–10 fold depending on which cancer cell line was used as a target [20]. In contrast, no to moderate decrease of activity upon loading into CB[7] has been reported for a dinuclear platinum complex [50]. The latter finding could be confirmed in an *in vivo* cancer model using Balb/c mice bearing human ovarian cancer [51]. Thus it seems that potential inhibitory effects of loading drugs into CB[7] have to be determined for each individual drug however overall there is good evidence that a variety of guest drug molecules will not significantly decrease in bioactivity upon binding to CB[7] containers.

## Materials and Methods

### Materials and Instruments

Trypsin/EDTA, Dextran-Alexa647, and Prolong® Gold Antifade Agent were purchased from Invitrogen. Erythromycin, erythromycin estolate and Hoechst33342 were purchased from Sigma-Aldrich. Camptothecin was obtained from Calbiochem. Instruments used included Spectramax M5e (Molecular Devices), Leica SP5 X Confocal, and BD FACSCanto II.

### Cell and Bacterial Culture

RAW264.7 cells (Mouse leukaemic monocyte macrophage, ATCC #TIB-71) and HEK 293 cells (Human Embryonic Kidney, ATCC #CRL-1573) were grown in DMEM (GIBCO®) media (Invitrogen) with 10% heat inactivated fetal calf serum (FCS) (Hyclone), 2% HEPES (Invitrogen), and 1% Penicillin/Streptomycin (Invitrogen). HepG2 (Heptacellular carcinoma, Human, ATCC #HB-8065) were grown in MEM media (Sigma) with 10% FCS, 2% HEPES, and 1% Penicillin/Streptomycin. *M. smegmatis* was cultured as described in Velmurugan *et al* [52].

### CB[n] Synthesis and Labeling with Fluorophores

Cucurbit[n]urils CB[7], CB[5], pentamer **1**, and FITC-spermine conjugate **4** were synthesized according to the published procedures [12], [24], [25]. The synthesis and recognition properties of compounds **2** and **3** will be reported separately [23].

#### Compound 1-(1-adamantyl)piperazine

A mixture of 1-bromoadamantane (2.00 g, 9.29 mmol) and piperazine (4.80 g, 55.7 mmol) were heated at 210°C in a pressure tube for 16 h. The reaction mixture was cooled to RT and dissolved in CHCl_3_ (60 mL). The organic layer was washed with 0.1 M NaOH (2×25 mL) and brine (2×25 mL) then concentrated. The crude solid was dissolved in EtOH (30 mL) followed by the addition of conc. HCl. After concentration, the solid was washed with EtOH (10 mL), centrifuged, the supernatant decanted, and the solid dried under high vacuum yielding 1-(1-adamantyl)piperazine dihydrochloride (1.66 g, 6.46 mmol, 69%) as an off-white solid. M.p. 299–301°C. IR (KBr, cm^−1^): 3456s, 3412s, 2920s, 2854s, 2712s, 2481s, 2423s, 1445 m, 1364s, 896 m, 650 m. ^1^H NMR (400 MHz, D_2_O): 3.57 (br. m, 8H), 2.27 (s, 3H), 1.99 (s, 6H), 1.75 (d, *J* = 12.0, 3H), 1.67 (d, *J* = 12.0, 3H). ^13^C NMR (100 MHz, CDCl_3_): 67.2, 41.9, 41.4, 36.0, 35.0, 29.7.

#### Compound 5

A stock solution (4.1 mM) of 4.06 mg 1-(1-adamantyl)piperazine in sodium bicarbonate buffer solution (4.5 mL, 100 mM, pH = 8.30) was prepared. A portion of this stock solution (0.234 mL, 9.6×10^−7^ mol) was added to 1.0 mg (8.0×10^−7^ mol) of the Alexa Fluor 555 carboxylic acid NHS ester (Invitrogen, Cat. #20009). The solution was stirred at room temperature for 1 h and then concentrated under high vacuum to yield **5** which was used without further purification. Compound **5** was stored as a stock solution in sodium phosphate buffer (0.5 mL, 50 mM, pH = 7.22).

#### CB[7] Complexes

Complexes CB[7]•**4** and CB[7]•**5** were prepared separately by dissolving CB[7] (0.44 mg, 0.32 µmol) in the stock solutions of **4** (0.2 mL, 1.6 mM) or **5** (0.2 mL, 1.6 mM) in sodium phosphate buffer (50 mM, pH = 7.22) at room temperature. The CB[7]•ethambutol complex was prepared by dissolving equimolar amounts of CB[7] and ethambutol in sodium phosphate buffer (50 mM, pH = 7.22) at room temperature.

### Cell Viability and Toxicity Assays

Cells were seeded in 96-well microtiter plates (Corning) at a concentration of 2.5×10^6^ cells/mL for HEK293 cells, 4×10^5^ cells/mL for HepG2 cells and 8×10^4^ cells/mL for RAW264.7 cells in 200 µL/well. After 24 hours, the HEK293 and RAW264.7 cells were treated with camptothecin (500 nM), erythromycin, erythromycin estolate, CB[7], CB[5], **1, 2**, or **3** at 10 µM, 100 µM, and 1 mM each. Six technical replicates were designated for untreated cells and four technical replicates were designated for the cells treated with camptothecin, erythromycin, erythromycin estolate and each of the five containers. We used the CellTiter 96 AQueous Kit® assay (Promega), an MTS-based assay, to quantify cell viability by measuring cellular metabolism. As a complementary assay we used the adenylate kinase (AK) release assay, Toxilight®BioAssay (Lonza), to quantify necrotic cell death. Both assays were performed according to the instructions provided by the vendor after 48 h of incubation. The supernatant from the samples used in the MTS assay were used in the AK assay.

The collected absorbance and relative luminescence data were normalized to percent cell viability (MTS) and percent cell death (AK) using equations 1 and 2:
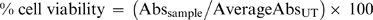
(1)

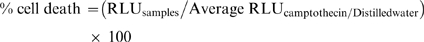
(2)


### Uptake Assay

RAW264.7 cells were seeded in a 24 well plate (Corning) at 5×10^5^ cells/mL. All samples were done in technical quadruplets and then combined to form doublets per sample. Controls included untreated cells and cells treated with **4** alone. The CB[7]•**4** complex was incubated with cells at the respective concentrations for 20 minutes and then collected for analysis. Cells were collected and fixed with 4% paraformaldehyde (PFA) (Fisher), in PBS (MediaTech Inc) before analysis by flow cytometry.

For the time course assay, CB[7]•**4** was again incubated with cells for 20 min. at a concentration of 32 µM after which time the cells were washed and incubated for 15, 20, 120 min. with infection medium (DMEM, and 10% FCS) before analysis by flow cytometry.

### Intracellular Localization

RAW264.7 cells were seeded at 10^4^ cells/well in 3-well glass slides (Electron Microscopy Sciences). Controls included cells stained with Hoechst33342 staining alone, Dextran-647 alone and CB[7]•**5** alone. Dextran-647 was incubated with cells overnight at a concentration of 125 µg/mL. The CB[7]•**5** complex was incubated with cells for 20 min. at a concentration of 32 µM the following day and then chased with infection medium for 15, 45, and 120 min. Before analysis, cells were fixed with 4% PFA, washed and immobilized with Prolong® Gold Antifade Agent (Invitrogen).

### Mycobacterial Killing Assay

RAW264.7 cells were seeded at 5×10^5^ cells/mL in one well of a 24-well plate. As controls we examined untreated cells on day 0 and day 3. Each sample was tested in technical duplicates. Cells were infected with *M. smegmatis* at a multiplicity of infection (MOI) of 10∶1 for 2 h and then incubated with chase media (infection media with varying concentrations of EMB or CB[7]•EMB) for 3 days. The EMB and CB7•EMB were used at minimum inhibitory concentration (MIC) values of 0.1 (2.4 µM), 0.4 (9.6 µM), 0.8 (19.2 µM) and 1 (24 µM) [53] units during *M. smegmatis* treatment. On day 3, cells were lysed with 1 ml/well of distilled water/0.05%Tween-80. The cell lysate for each condition was added to 7H9 (Difco® Middlebrook) media. These solutions were then serially diluted four times and plated in technical triplicates of 5 µL each on 7H10 (Difco® Middlebrook) agar plates. Viable bacteria were quantified by calculating the number of colony forming units (CFU) per mL.

### Statistical Analysis

Statistical analysis of data was performed using the unpaired t-test at 95% confidence interval (*P = 0.01–0.05; **P = 0.001–0.01; ***P<0.001). All data were plotted using mean ± SEM excluding Figure 6 which was graphed with mean ± SD. For all experiments, one representative figure out of at least three independent experiments is shown unless indicated otherwise.
